# Predictive values of various serum biomarkers in women with suspected preeclampsia: A prospective study

**DOI:** 10.1002/jcla.23740

**Published:** 2021-02-22

**Authors:** Jing Wang, Honghai Hu, Xiaowei Liu, Shenglong Zhao, Yuanyuan Zheng, Zhaoxia Jia, Lu Chen, Chunhong Zhang, Xin Xie, Junhui Zhong, Ying Dong, Jingrui Liu, Yifan Lu, Zhen Zhao, Yanhong Zhai, Juan Zhao, Zheng Cao

**Affiliations:** ^1^ Department of Laboratory Medicine Beijing Obstetrics and Gynecology Hospital Capital Medical University Beijing China; ^2^ Guangzhou Kangrun Biotech Co. Ltd Guangdong China; ^3^ Department of Obstetrics Beijing Obstetrics and Gynecology Hospital Capital Medical University Beijing China; ^4^ Department of Information and Statistics Beijing Obstetrics and Gynecology Hospital Capital Medical University Beijing China; ^5^ Scientific & Application Division Sysmex Shanghai Ltd Beijing China; ^6^ Department of Pathology and Laboratory Medicine Weill Cornell Medicine New York NY USA; ^7^ Hospital Administration Office Beijing Obstetrics and Gynecology Hospital Capital Medical University Beijing China

**Keywords:** cohort, prediction, preeclampsia, prospective, serum biomarker

## Abstract

**Background:**

Preeclampsia (PE) prediction has been shown to improve the maternal and fetal outcomes in pregnancy. We aimed to evaluate the PE prediction values of a series of serum biomarkers.

**Methods:**

The singleton pregnant women (20–36 gestational weeks) with PE‐related clinical and/or laboratory presentations were recruited and had the blood drawn at their first visits. The following markers were tested with the collected serum samples: soluble fms‐like tyrosine kinase 1 (sFlt‐1), placental growth factor (PlGF), thrombomodulin (TM), tissue plasminogen activator inhibitor complex (tPAI‐C), complement factors C1q, B, H, glycosylated fibronectin (GlyFn), pregnancy‐associated plasma protein‐A2 (PAPP‐A2), blood urea nitrogen (BUN), creatinine (Cre), uric acid (UA), and cystatin C (Cysc).

**Results:**

Of the 196 recruited subjects, 25% (*n* = 49) developed preeclampsia before delivery, and 75% remained preeclampsia negative (*n* = 147). The serum levels of sFlt‐1, BUN, Cre, UA, Cysc, and PAPP‐A2 were significantly elevated, and the PlGF level was significantly decreased in the preeclampsia‐positive patients. In the receiver operating characteristics (ROC) analyses, the area under the curves were listed in the order of decreasing values: 0.73 (UA), 0.67 (sFlt‐1/PlGF), 0.66 (Cysc), 0.65 (GlyFn/PlGF), 0.64 (PAPP‐A2/PlGF), 0.63 (BUN), 0.63 (Cre), and 0.60 (PAPP‐A2). The positive predictive values of these serum markers were between 33.1% and 58.5%, and the negative predictive values were between 80.9% and 89.5%.

**Conclusions:**

The serum markers investigated in current study showed better performance in ruling out than ruling in PE. Absence of pre‐defined latency period between blood draw and the onset of PE limits the clinical utility of these markers.

## Graphical abstract

In present study, we evaluated the predictive values of the following serum biomarkers in a prospective study with the women suspected to develop preeclampsia (PE): soluble fms‐like tyrosine kinase 1 (sFlt‐1), placental growth factor (PlGF), pregnancy‐associated plasma protein‐A2 (PAPP‐A2), glycosylated fibronectin (GlyFn), thrombomodulin (TM), tissue plasminogen activator inhibitor complex (tPAI‐C), uric acid (UA), blood urea nitrogen (BUN), creatinine (Cre), and cystatin C (Cysc). The graphical image showed the ROC analyses of the serum markers for PE prediction in the prospective cohort with PE‐related clinical or laboratory presentations. A. ROC analyses for sFlt‐1 and sFlt‐1/PlGF; B. BUN, Cre, UA, and Cysc; C. PAPP‐A2, GlyFn/PlGF and PAPP‐A2/PlGF; D. PlGF.

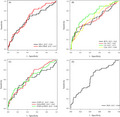

## 1 INTRODUCTION

Preeclampsia, one of the most common complications during pregnancy, is estimated to have an incidence rate of 2–8% worldwide^1^ and can lead to serious maternal or fetal morbidity and mortality if not managed properly.^2^ Extensive studies have been performed to reveal the clinical value of preeclampsia prediction,^3^ of which three major beneficial effects may be concluded: identifying high‐risk patients who require close monitoring to decrease potential complications, reducing the necessity of antenatal care of low‐risk populations, and promoting the development of early therapeutic interventions of preeclampsia.^4^

According to a systematic review published in 2019 about preeclampsia prediction models,^5^ most previous studies (87%) have conducted risk assessments during the first trimester of pregnancy. In first trimester preeclampsia screening, the prediction model is recommended to combine maternal background risk factors, imaging tests, and serum biomarkers to increase sensitivity and reduce the false detection rate.^5^ Due to relatively low positive predictive values (PPV) (8–33%) during first trimester screening for preeclampsia,^6^ false‐positive patients who do not develop preeclampsia may be exposed to unnecessary tests and prophylactic interventions with no benefit.

Recently, some researchers evaluated preeclampsia predictive markers in patient groups >20 gestational weeks and identified high‐risk factors or clinical/laboratory signs of preeclampsia.^7^, ^8^ This type of testing scenario with patients suspected of preeclampsia development has been proven effective, especially in the studies on soluble fms‐like tyrosine kinase‐1 (sFlt‐1) and placental growth factor (PlGF).^7^, ^9^ For example, a sFlt‐1/PlGF ratio of 38 was found to effectively exclude preeclampsia with a negative predictive value (NPV) of 99.3%^7^; however, a similar prospective study with Chinese pregnant women has yet to be published. Interestingly, a recent longitudinal study conducted in Singapore suggested significant differences in the PlGF and sFlt‐1 concentrations during pregnancy between different Asian ethnicities (Chinese, Malay, and Indian).^10^ In a retrospective study with 118 Chinese singleton pregnancies who had been diagnosed with preeclampsia, the sFlt‐1/PlGF ratio was shown to be an efficient marker in differentiating preeclampsia and predicting the timing of delivery for preeclampsia pregnancies.^11^

In addition to sFlt‐1/PlGF, a series of other serum biomarkers have been shown to be associated with the occurrence or outcomes of preeclampsia. For instance, the maternal pregnancy‐associated plasma protein‐A2 (PAPP‐A2) serum concentration was found to be upregulated in preeclampsia patients, resulting in local activation of insulin‐like growth factor (IGF) signaling pathways.^12^ This finding implied that PAPP‐A2 may be upregulated in preeclampsia to compensate for IGF binding protein 5‐mediated pathway.^12^ The maternal serum glycosylated fibronectin (GlyFn) was reported to be elevated in all three trimesters of preeclampsia patients; the test was further recommended as a point‐of‐care biomarker to quickly determine risk for preeclampsia and for poor maternal and fetal outcomes among preeclamptic patients.^13^ In uteroplacental thrombosis, which is one of the major mechanisms of preeclampsia, several thrombotic and fibrinolytic factors including circulating soluble thrombomodulin (TM) and tissue plasminogen activator (tPA) were found to be elevated in PE and correlated with the severity of proteinuria.^14^, ^15^ The relative changes of these coagulation factors reflected endothelial disturbance in preeclampsia, and they were recommended for future evaluation as potential risk biomarkers.^14^, ^15^ The dysregulation of complement pathways also contributes to the development of preeclampsia. The differential expression of complement factors C1q, B, and H were found in specific trimesters of severe preeclampsia patients, suggesting promising values as diagnostic markers for severe preeclampsia.^16^ The presence of proteinuria, which is a hallmark in preeclampsia, indicates that renal deficiency contributes significantly to the pathogenesis of preeclampsia.^17^ The renal function markers, such as uric acid (UA), blood urea nitrogen (BUN), creatinine (Cre), and cystatin C (Cysc), have been found to be disturbed in preeclampsia patients^17^ and their performance in predicting preeclampsia after 20 weeks of gestation still lacks validation studies.

In summary, even though the panel of serum markers described above have been studied in a broad context of preeclampsia, whether or not these biomarkers will add value in preeclampsia prediction remains largely unknown. In this work, we aimed to evaluate the predictive values of: (1) the known markers of sFlt‐1 and PlGF, and (2) PAPP‐A2, GlyFn, TM, tissue plasminogen activator inhibitor complex (tPAI‐C), complement factors C1q, B, and H, and renal function markers including UA, BUN, Cre, and Cysc, in a prospective study with Chinese pregnant women who were suspected to develop preeclampsia.

## 2 MATERIALS AND METHODS

### 2.1 Subjects

The enrollment criteria for women with suspected preeclampsia are described as follows. The recruited singleton pregnant women were at least 18 years old and between 20 and 36 gestational weeks (GWs), as pregnancies of >36 GWs are likely to be subjected to delivery of fetus if preeclampsia is diagnosed or the blood pressure (BP) is severely elevated. In addition, one of the following recruiting criteria had to be met for patient enrollment: new onset of hypertension (systolic BP >120 and <160 mmHg and/or diastolic BP >80 and <110 mmHg) or proteinuria (≥2+ by dipstick); aggravation of preexisting hypertension or proteinuria; or persistent symptoms of upper abdominal pain, edema, visual impairment, abnormal weight gain (>1 kg/week), decreased platelets (<150 × 10^9^/L), elevated liver transaminase (alanine transferase >55 U/L or aspartate transaminase >34 U/L), fetal growth restriction (estimated fetal weight or abdominal circumference <10th percentile according to the charts routinely used by Obstetric Department at our institute), increased pulsatility index (PI) of the uterine artery (PI > 0.878), or uterine artery flow notching. The subjects meeting one of the following conditions were excluded confirmed diagnosis of preeclampsia or hemolysis elevated liver enzymes and low platelets (HELLP) syndrome or anti‐hypertensive treatment during this pregnancy. The recruited pregnant subjects had their blood drawn at their first visits to Beijing Obstetrics and Gynecology Hospital, with follow‐up for the presence (“preeclampsia‐positive” group) or absence (“preeclampsia‐negative” group) of preeclampsia until delivery.

The preeclampsia diagnosis was determined with the diagnostic criteria proposed by the 2019 ACOG Practice Bulletin,^6^ in which preeclampsia was defined as gestational hypertension (systolic/diastolic blood pressure ≥140/90 mmHg) in previously normotensive women accompanied by proteinuria (urine protein ≥300 mg/24 h) or end‐organ damage after 20 weeks of gestation.

### 2.2 Serum samples, reagents, and methods

The maternal blood from each participant (3 mL) was drawn when they were enrolled and left to clot for 30 min followed by centrifugation for 10 min at 2300 *g*. The serum aliquots (1 mL) were separated and stored at −80°C until being tested.

The maternal levels of sFlt‐1 (Cat No. YZB/GER5424‐2014, Roche Diagnostics) and PlGF (Cat No. YZB/GER5425‐2014, Roche Diagnostics) were measured on the fully automated electrochemiluminescence immunoassay platform COBAS e411 (Roche Diagnostics). Maternal serum PAPP‐A2 (Cat No. AL109, Ansh Labs) and GlyFn (Cat No. AL160, Ansh Labs) levels were determined with single measurement by the enzyme‐linked immunosorbent assay (ELISA) according to the manufacturer's instructions. The total coefficients of variation (CVs) for PAPP‐A2 and GlyFn were 4.1–4.7% and 3.2–3.4%, respectively. The ELISA standard operation protocol was performed as previously described.^5^, ^18^ The serum TM (HISCL^®^ TM Assay Kit, Sysmex) and tPAI‐C (HISCL^®^ tPAI‐C Assay Kit, Sysmex) levels were determined by the fully automated HISCL‐5000 Chemiluminescence Analyzer (Sysmex). The complement factors C1q (Cat No. 20170922, Shanghai Beijia Biochemical Reagent), B (Cat No. 20020803, Shanghai Beijia Biochemical Reagent), and H (Cat No. 20183020, Shanghai Beijia Biochemical Reagent) levels were measured using a fully automated ARCHITECT ci16200 Integrated System Chemistry/Immunology Analyzer (Abbott). The renal function tests including UA (Cat No. 3P39‐21, Abbott), BUN (Cat No. 7D75‐21, Abbott), Cre (Cat No. 8L24‐31, Abbott), and Cysc (Cystatin C Assay Kit, Beijing Jiuqiang Biotech) were also performed on the ARCHITECT ci16200 Analyzer (Abbott).

### 2.3 Statistical analysis

Data analysis was performed using statistical software SPSS 23.0. The Kolmogorov–Smirnov test was used to evaluate the normality of the data distribution. Numerical values were expressed as the mean and standard deviation (SD) for variables with normal distribution and as the median and percentiles for nonnormally distributed data. Comparisons between the two groups were performed using the *t* test (for normal distribution) or Mann–Whitney *U* test (for non‐normal distribution). Categorical variables were expressed as frequencies and proportion; comparisons between the two groups were tested by chi‐square test. The receiver operating characteristics (ROC) curve was used to analyze the predictive values of the markers for preeclampsia. The comparison of before and after adjusted area under curves (AUCs) was assessed using the algorithm developed by DeLong et al.^19^ The adjusted factors included age, prepregnancy BMI, parity, and underlying chronic diseases in the ROC analyses. Sensitivity, specificity, and cutoff values were reported when Youden's index was at the maximum or specificity was fixed at 90%. A binary logistic regression analysis was performed including age, prepregnancy BMI, parity, underlying chronic diseases, and each of the markers. All statistical tests were two‐sided, and *p* < 0.05 was considered statistically significant.

## 3 RESULTS

**FIGURE 1:**
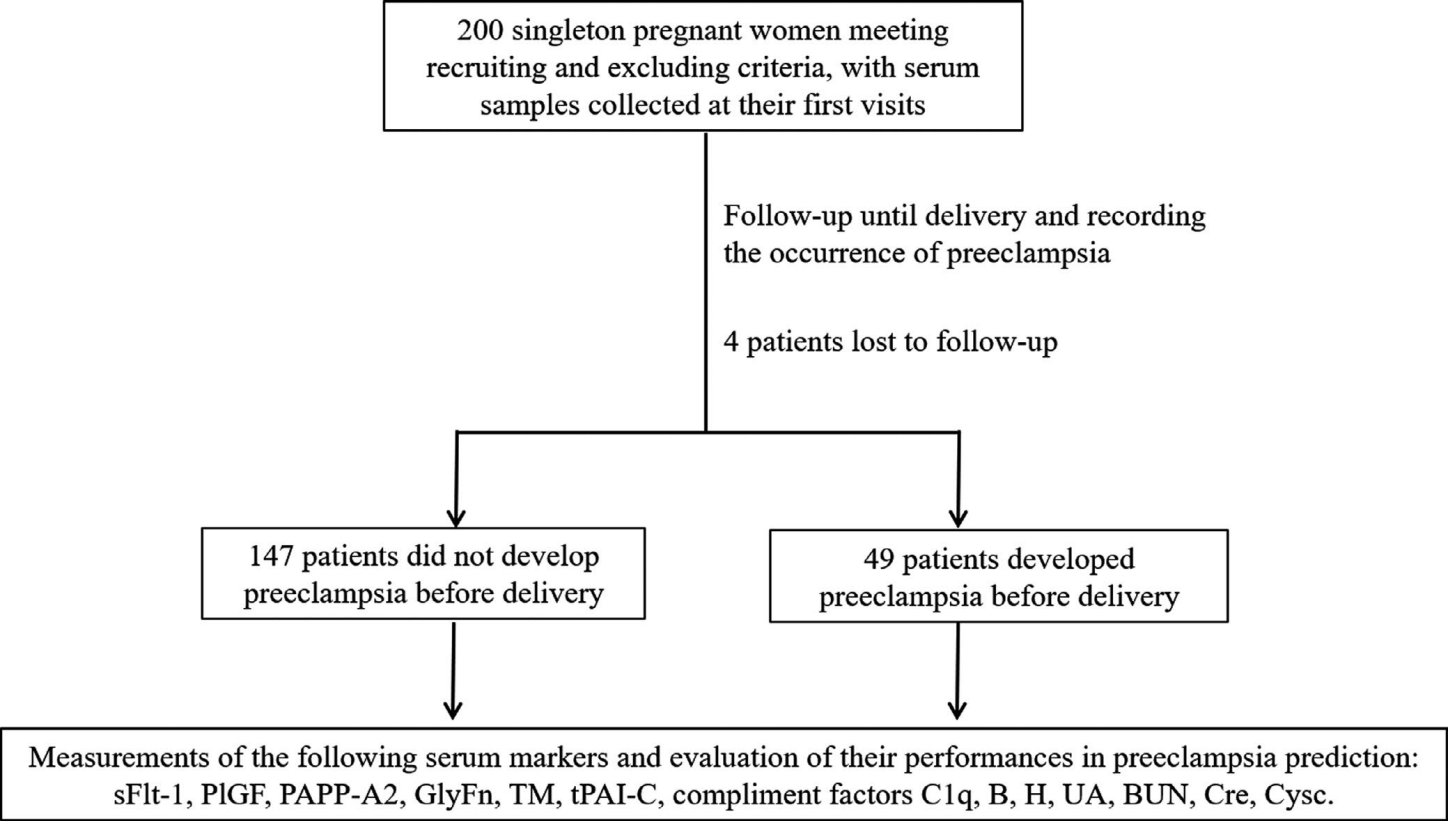
Schematic diagram depicting patient recruitment and study design. BUN, blood urea nitrogen; Cre, creatinine; Cysc, cystatin C; GlyFn, glycosylated fibronectin; PAPP‐A2, pregnancy‐associated plasma protein‐A2; PlGF, placental growth factor; sFlt‐1, soluble fms‐like tyrosine kinase 1; TM, thrombomodulin; tPAI‐C, tissue plasminogen activator inhibitor complex; UA, uric acid

A flowchart depicting patient recruitment and the study design is presented in Figure 1. From January 2018 to March 2019, a total of 200 subjects with preeclampsia‐related clinical and/or laboratory presentations were recruited, including four patients who were lost to follow‐up. Of the remaining 196 patients, 25% (49/196) (preeclampsia‐positive group) developed preeclampsia before delivery, and 75% (147/196) (preeclampsia‐negative group) maintained preeclampsia negative for remainder of the pregnancy. Since the focus was narrowed down on the patients with relevant symptoms, the prevalence of preeclampsia (25%) in the current cohort was much higher than that of the overall pregnancies (5.2%) during the study period in our institute. The demographic data of all the recruited subjects are available in Table S1. As shown in Table S2, no significant difference was found between the preeclampsia‐positive and negative groups for the main symptoms identified in the enrolled patients (new onset of hypertension or aggravation of preexisting hypertension, new onset of proteinuria or aggravation of preexisting proteinuria, edema, and fetal growth restriction).

The collected serum samples of the preeclampsia‐positive (*n* = 49) and preeclampsia‐negative (*n* = 147) patients were subjected to the following serum marker measurements: sFlt‐1; PlGF; PAPP‐A2; GlyFn; TM; tPAI‐C; complement factors C1q, B, and H; UA; BUN; Cre; and Cysc. As summarized in Table 1, except for underlying chronic diseases, no significant difference was found in maternal age, prepregnancy BMI, blood sampling gestational weeks (GW), gravidity, or parity between the two groups; however, patients with underlying diseases (hypertension being the most prominent) that are associated with preeclampsia development were more likely to develop preeclampsia during pregnancy (*p* = 0.010, Table 1). On average, the time interval from serum collection to preeclampsia occurrence was 7 weeks in the preeclampsia‐positive patients (Table 1).

**TABLE 1:** Demographic data for the recruited subjects

	Preeclampsia negative (*n* = 147)	Preeclampsia positive (*n* = 49)	*p* Value
Age^a^ (years)	33 (29–36)	34 (31–37)	0.066
Prepregnancy BMI^a^ (kg/m^2^)	23.6 (21.2–25.9)	23.2 (20.7–28.1)	0.915
Sampling GW^a^ (weeks)	29 (24–33)	30 (25–32)	0.702
Preeclampsia diagnosis GW^a^ (weeks)	Not applicable	37 (34–38)	
Gravidity^b^			
≥3	67.8% (99)	59.2% (29)	0.271
<3	32.2% (47)	40.8% (20)	
Parity^b^			
0	74.0% (108)	61.2% (30)	0.090
≥1	26.0% (38)	38.8% (19)	
Underlying chronic disease^b^			
No	84.4% (124)	67.3% (33)	0.010
Yes	15.6% (23)	32.7% (16)	
Hypertension	6.8% (10)	26.5% (13)	
Hypothyroidism or hyperthyroidism	2.7% (4)	8.2% (4)	
Gestational diabetes mellitus	3.4% (5)	0	
Polycystic ovary syndrome	2.7% (4)	0	
Antiphospholipid syndrome	0.7% (1)	0	

^a^ Age, prepregnancy BMI, sampling GW (gestational week), preeclampsia diagnosis GW were presented as median (25th–75th percentile).

^b^ The underlying chronic diseases that may be considered as risk factors for preeclampsia development were presented as percentages. The number in the parentheses after percentage figures indicated the number of the subjects that had the corresponding underlying chronic disease when they were enrolled.

**FIGURE 2:**
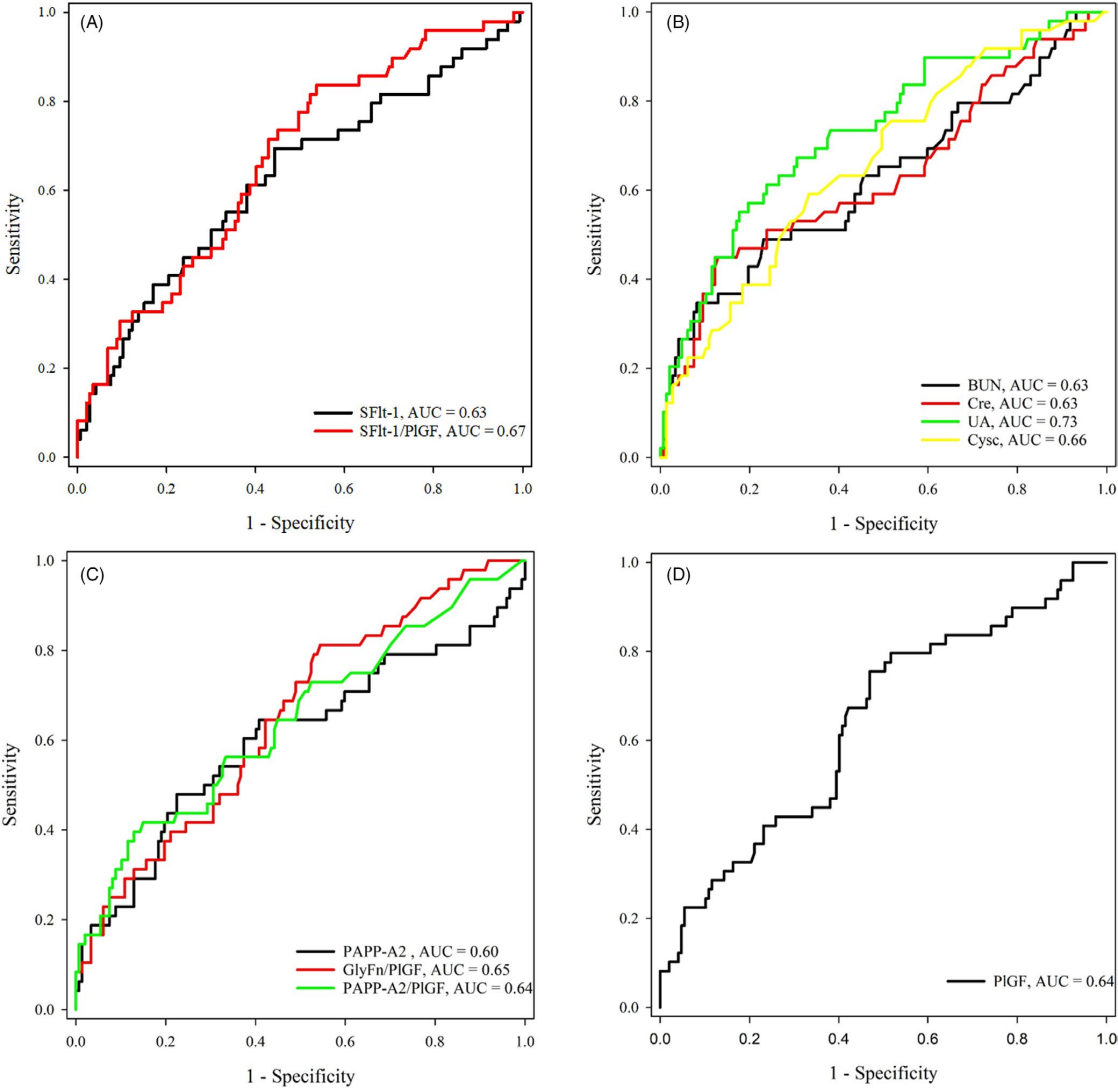
ROC analyses of the serum markers for PE prediction in the prospective cohort with PE‐related clinical or laboratory presentations. (A) ROC analyses for sFlt‐1 (AUC = 0.63) and sFlt‐1/PlGF (AUC = 0.67); (B) BUN (AUC = 0.63), Cre (AUC = 0.63), UA (AUC = 0.73), and Cysc (AUC = 0.66); (C) PAPP‐A2 (AUC = 0.60), GlyFn/PlGF (AUC = 0.65), and PAPP‐A2/PlGF (AUC = 0.64); (D) PlGF (AUC = 0.64)

To evaluate the preeclampsia predictive values of the selected markers in present study, we first compared their serum concentrations that were determined using our laboratory devices and platforms. As shown in Table 2, a panel of analytes representing various biological functions were found to be significantly elevated in the patients who developed preeclampsia later in pregnancy compared to the preeclampsia‐negative group, including sFlt‐1 (*p* = 0.007), BUN (*p* = 0.009), Cre (*p* = 0.006), UA (*p* < 0.001), Cysc (*p* = 0.001), and PAPP‐A2 (*p* = 0.032). The PlGF level (*p* = 0.004) was the only marker that was significantly decreased in the preeclampsia‐positive patients, resulting in more profoundly increased calculated ratios, including sFlt‐1/PlGF (*p* < 0.001), GlyFn/PlGF (*p* = 0.002), and PAPP‐A2/PlGF (*p* = 0.003). By contrast, the hemostatic factors (TM and tPAI‐C) and the complement factors (C1q, B, and H) were not significantly different between the preeclampsia‐negative and positive groups. The significantly changed serum markers either from direct laboratory measurements or from the ratio calculations were then subjected to ROC analyses. As shown in Figure 2 and Table 3, the AUCs were listed in the order of decreasing values: 0.73 (UA), 0.67 (sFlt‐1/PlGF), 0.66 (Cysc), 0.65 (GlyFn/PlGF), 0.64 (PlGF), 0.64 (PAPP‐A2/PlGF), 0.63 (sFlt‐1), 0.63 (BUN), 0.63 (Cre), and 0.60 (PAPP‐A2). After adjusted for age, prepregnancy BMI, parity and underlying chronic diseases, the improvement of the prediction power of each serum marker was insignificant (*p* > or = 0.05) (Table 3).

**TABLE 2:** Comparison of serum predictors in the preeclampsia‐positive and preeclampsia‐negative groups

	Preeclampsia negative^a^ (*n* = 147)	Preeclampsia positive^a^ (*n* = 49)	*p* Value
sFlt‐1 (pg/mL)	2036 (1548–3113)	2814 (1785–4800)	0.007
PlGF (pg/mL)	301.4 (135.7–511.9)	209.1 (69.5–293.3)	0.004
sFlt‐1/PlGF	6.8 (3.6–21.7)	13.3 (6.8–65.0)	<0.001
TM (IU/mL)	9.9 (8.7–10.2)	10.7 (8.8–12.3	0.405
tPAI‐C (ng/mL)	5.9 (4.5–7.3)	5.4 (3.7–6.8)	0.154
BUN (mmol/L)	2.8 (2.4–3.4)	3.3 (2.5–4.3)	0.009
Cre (μmol/L)	40.8 (36.8–44.8)	45.4 (38.1–51.1)	0.006
UA (μmol/L)	232.3 (201.5–280.1)	295.7 (233.2–336.0)	<0.001
Cysc (mg/mL)	0.9 (0.8–1.1)	1.1 (0.9–1.4)	0.001
C1q (mg/L)	204.0 (177.0–228.0)	194.0 (170.0–226.0)	0.346
B factor (mg/L)	333.0 (308.0–365.0)	353.5 (328.5–369.3)	0.036
H factor (mg/L)	404.0 (372.0–429.0)	397.5 (358.0–436.8)	0.664
GlyFn (mg/mL)	259.1 (220.1–323.3)	285.4 (227.1–421.9)	0.061
PAPP‐A2 (mg/mL)	52.9 (31.4–103.7)	91.8 (38.3–192.7)	0.032
GlyFn/PlGF	0.9 (0.5–2.3)	1.5 (0.8–6.0)	0.002
PAPP‐A2/PlGF	0.2 (0.1–0.7)	0.5 (0.1–2.3)	0.003

^a^ Presented as median (25th–75th percentile).

**TABLE 3:** Comparison of AUCs before and after adjusted for demographic data of recruited subjects

	Unadjusted AUC (95% CI)	Adjusted^a^ AUC (95% CI)	*p* Value^b^
sFlt‐1/PlGF	0.67 (0.59– 0.73)	0.70 (0.63–0.77)	0.457
BUN (mmol/L)	0.63 (0.56–0.70)	0.70 (0.62–0.76)	0.050
Cre (μmol/L)	0.63 (0.57– 0.72)	0.73 (0.66–0.79)	0.057
UA (μmol/L)	0.73 (0.66–0.79)	0.77 (0.70–0.83)	0.104
Cysc (mg/mL)	0.66 (0.59–0.73)	0.70 (0.63–0.76)	0.312
GlyFn/PlGF	0.65 (0.57–0.71)	0.70 (0.63–0.76)	0.225
PAPP‐A2/PlGF	0.64 (0.57–0.71)	0.72 (0.65–0.79)	0.058

Abbreviations: AUC, area under the curves; CI, confidence interval.

^a^ Adjusted for age, prepregnancy BMI, parity and underlying chronic disease.

^b^ Comparison *p* valued before and after adjusted AUC.

The serum marker measurements were subsequently analyzed by binary logistic regression analysis, with age, prepregnancy BMI, parity, and underlying chronic diseases as covariates. The logistic regression analysis showed that all the listed serum markers were independent risk factors (*p* < 0.05) for preeclampsia development, with Cysc, BUN and PAPP‐2/PlGF having the highest OR values (Table S3). With the cutoff values obtained with the highest Youden index (sum of sensitivity and specificity minus one) in the ROC analyses, the PPVs were between 33.1% and 58.5%, and the NPVs were between 80.9% and 89.5% (Table 4); with specificity fixed at 90%, the serum makers with the highest sensitivities were Cre (36.7%), UA (34.7%), and BUN (34.7%) (Table S4).

**TABLE 4:** The performances of serum biomarkers in predicting preeclampsia

	Cutoff value	PPV^a^ (%)	NPV^b^ (%)
sFlt‐1/PlGF	5.6	34.0	89.5
BUN (mmol/L)	3.9	58.5	80.9
Cre (μmol/L)	48.0	53.5	82.7
UA (μmol/L)	280.7	46.0	85.6
Cysc (mg/mL)	1.0	37.1	83.1
GlyFn/PlGF	0.7	33.1	88.0
PAPP‐A2/PlGF	1.0	48.0	81.5

^a^ Positive predictive value.

^b^ Negative predictive value.

## 4 DISCUSSION

Although large amount of research has been focused on preeclampsia prediction during pregnancy, very few serum prediction markers have been successfully implemented in clinical practice. With the low prevalence of preeclampsia in the general pregnant population, the application of specific laboratory test(s) would be costly to apply universally during pregnancy. In the publication for evaluating the preeclampsia predictive value of sFlt‐1/PlGF by Zeisler et al.,^7^ the authors narrowed down the targeting patients who presented with preeclampsia‐related clinical and/or laboratory presentations. A similar patient recruiting strategy was adopted in our study. With a narrow focus on the subgroup of patients more likely to develop preeclampsia, medical resources may be better directed at high‐risk patients; however, unlike universal screening, stratifying pregnant women based on their clinical symptoms and/or usual laboratory findings certainly requires extra effort. Whether an economic benefit exists in the overall management of preeclampsia remains a question.

A 4‐week observation window along with the cutoff value of 38 was applied in the article by Zeisler et al.,^7^ which showed that the sFlt‐1/PlGF ratio could accurately exclude preeclampsia occurrence in suspicious patients, with an AUC of 0.90 in the ROC analysis compared to an AUC of 0.67 in our study with follow‐up until delivery; however, for the remaining markers included in present study, the observation window was yet to be well‐defined; delivery remained the mainstream endpoint in most of the preeclampsia prediction studies.^5^, ^20^ The average interval between blood sampling and preeclampsia diagnosis was 7 weeks with our prospective cohort, which provided important clinical evidence for future validation studies. Interestingly, with the previously reported cutoff value of 38 for the sFlt‐1/PlGF ratio and 4‐week observation window, only 15 recruited subjects developed preeclampsia in our study, which was 30.5% (15/49) of the total preeclampsia‐positive patients (Table S5). Moreover, the NPV (94.4%) was close to that previously reported,^7^ the sensitivity (40.0%), specificity (83.4%), and PPV (16.7%) were much lower with our cohort (Table S5), suggesting that ethnicity may be a confounding factor for the application of the sFlt‐1/PlGF ratio and the cutoff value needs to be further optimized for Chinese populations before clinical implementation.

The hemostatic factors such as TM and tPAI‐C were found to be related to the incidence and severity of PE decades ago.^21^ In preeclampsia patients, significant endothelial disturbance and procoagulant potential, along with aberrant expression of these hemostatic factors, were reported in previous studies^14^, ^15^; however, whether they could be useful in preeclampsia prediction has yet to be investigated. With our cohort, no difference was observed between the preeclampsia‐positive and negative groups, indicating their limited values in preeclampsia prediction (Table 2).

The excessive activation and poor regulation of the complement system at the maternal‐fetal interface contributes to the development of preeclampsia.^22^ More importantly, a recent study by Jia et al. showed that the complement factors C1q, B, and H were able to diagnose early‐onset severe preeclampsia with AUCs of 0.81, 0.74, and 0.68, respectively. To further evaluate their potential utility in preeclampsia prediction, the circulating levels were measured in the present study. No significant difference was found between the preeclampsia‐positive and preeclampsia‐negative groups (Table 2).

The two glycoproteins that were included in our testing panel, GlyFn and PAPP‐A2, have been widely studied in preeclampsia. In a 2020 study by Huhn et al.,^8^ the GlyFn level in a prospective cohort identified with preeclampsia‐specific high‐risk factors was reported to show satisfactory preeclampsia prediction with an AUC of 0.94 in the ROC analysis. In our study, GlyFn was also increased, although not significantly, in the patient group that developed preeclampsia. This apparent discrepancy may be introduced by differences in the sample size, patient recruiting criteria or testing methodology of the two studies. The glycoprotein PAPP‐A2, involved in cleaving insulin‐like growth factor binding protein in the placenta, was found to be helpful in diagnosing^12^ and predicting preeclampsia.^8^ In our study, the PAPP‐A2/PlGF ratio (*p* = 0.003) was found to be a better marker than PAPP‐A2 (*p* = 0.032) alone (Table 2), with an adjusted AUC of 0.72 (Table 3).

The common renal function tests such as BUN, Cre, UA, and Cysc were shown to be potentially valuable in preeclampsia diagnosis and prediction. For example, the BUN^23^ and BUN/Cre ratio ^24^ were both found increased in the preeclampsia patients compared with those in the normal controls. Cysc was also found to be elevated in preeclampsia patients^25^ and was able to predict preeclampsia in combination with neutrophil gelatinase‐associated lipocalin (AUC = 0.88).^26^ In a prospective study with a relatively large cohort (*n* = 9522) by Rezk et al.,^27^ the serum UA level during the second trimester was found to be a useful preeclampsia predictor for women at moderate or low risk. More interestingly, an elevated UA level was later reported to be a risk factor for women with gestational hypertension to develop preeclampsia and deliver small‐for‐gestational‐age infants.^28^ We observed similar findings in which UA was the most promising predictor with the greatest AUCs (0.73 and 0.77, before and after adjustment, respectively) in the ROC analyses (Figure 2 and Table 3).

In conclusion, in a prospective cohort suspected of preeclampsia development, the angiogenic modulators sFlt‐1 and PlGF; the renal function markers BUN, Cre, UA, and Cysc; and the glycoprotein PAPP‐A2 were significantly altered between the two groups. Last but not least, the serum markers invested in our study showed better performance in ruling out than ruling in preeclampsia. Absence of pre‐defined latency period between blood draw and the onset of preeclampsia or delivery significantly limited the clinical utility of these markers.

## CONFLICT OF INTEREST

The authors declare that they have no conflict of interest.

## AUTHOR CONTRIBUTIONS

All authors have certified the author list and the contribution description. All authors have read and approved the submitted manuscript and any substantially modified version of the manuscript. Contribution to work: J.W., H.H., X.L., S.Z., Y.Z., J.Z., Z.Z., Y.Z., J.Z., and Z.C. were involved in study conception and design and patient recruitment; L.C., C.Z., and X.X. were involved in performing the experiments, and data acquisition, analysis, and interpretation; H.H. and Z.C. drafted the article and critically reviewed and approved the final article; Z.J., Y.D., J.L., and Y.L. contributed to the statistical analysis and figure preparation.

## Supporting information

Table S1‐S5

## Data Availability

The baseline characteristics of the recruited subjects are provided as Table S1. Alternatively, Table S1 is available in the Open Science Framework Repository (www.osf.io, https://doi.org/10.17605/OSF.IO/JB5A7). According to the patients’ verbal consents, the raw testing results are only available from the corresponding author on reasonable request.
